# Identification of microRNAs responsive to arbuscular mycorrhizal fungi in *Panicum virgatum* (switchgrass)

**DOI:** 10.1186/s12864-022-08797-x

**Published:** 2022-10-05

**Authors:** Alex C. Johnson, Thomas H. Pendergast, Srinivasa Chaluvadi, Jeffrey L. Bennetzen, Katrien M. Devos

**Affiliations:** 1 Department of Plant Biology, University of Georgia, Athens, GA 30602 USA; 2 Institute of Plant Breeding, Genetics and Genomics (Department of Crop and Soil Sciences), University of Georgia, Athens, GA 30602 USA; 3 Department of Genetics, University of Georgia, Athens, GA 30602 USA

**Keywords:** microRNA, Arbuscular mycorrhizae, Switchgrass, *Panicum virgatum*

## Abstract

**Background:**

MicroRNAs (miRNAs) are important post-transcriptional regulators involved in the control of a range of processes, including symbiotic interactions in plants. MiRNA involvement in arbuscular mycorrhizae (AM) symbiosis has been mainly studied in model species, and our study is the first to analyze global miRNA expression in the roots of AM colonized switchgrass (*Panicum virgatum*), an emerging biofuel feedstock. AM symbiosis helps plants gain mineral nutrition from the soil and may enhance switchgrass biomass production on marginal lands. Our goals were to identify miRNAs and their corresponding target genes that are controlling AM symbiosis in switchgrass.

**Results:**

Through genome-wide analysis of next-generation miRNA sequencing reads generated from switchgrass roots, we identified 122 mature miRNAs, including 28 novel miRNAs. By comparing miRNA expression profiles of AM-inoculated and control switchgrass roots, we identified 15 AM-responsive miRNAs across lowland accession “Alamo”, upland accession “Dacotah”, and two upland/lowland F_1_ hybrids. We used degradome sequencing to identify target genes of the AM-responsive miRNAs revealing targets of miRNAs residing on both K and N subgenomes. Notably, genes involved in copper ion binding were targeted by downregulated miRNAs, while upregulated miRNAs mainly targeted GRAS family transcription factors.

**Conclusion:**

Through miRNA analysis and degradome sequencing, we revealed that both upland and lowland switchgrass genotypes as well as upland-lowland hybrids respond to AM by altering miRNA expression. We demonstrated complex GRAS transcription factor regulation by the miR171 family, with some miR171 family members being AM responsive while others remained static. Copper miRNA downregulation was common amongst the genotypes tested and we identified superoxide dismutases and laccases as targets, suggesting that these Cu-miRNAs are likely involved in ROS detoxification and lignin deposition, respectively. Other prominent targets of the Cu miRNAs were blue copper proteins. Overall, the potential effect of AM colonization on lignin deposition pathways in this biofuel crop highlights the importance of considering AM and miRNA in future biofuel crop development strategies.

**Supplementary Information:**

The online version contains supplementary material available at 10.1186/s12864-022-08797-x.

## Background

Plant microRNAs (miRNAs) are 21–22 nucleotide RNAs that are important post-transcriptional regulators of mRNA. They are derived from microRNA (*MIR*) genes [1], which are transcribed into pre-miRNAs by RNA polymerase II [2]. The pre-miRNAs contain regions of self-complementarity that form a stem-loop structure [3]. The double stranded portion of the stem-loop is recognized and cleaved by DICER LIKE PROTEIN 1 (DCL1), resulting in a double-stranded miRNA-miRNA* complex that is then exported to the cytoplasm [3, 4]. There, the mature miRNA is separated from its complement, miRNA*, which, in most cases, is degraded. However, there have been reports of gene silencing by miRNA* [5]. The mature miRNA is then loaded onto ARGONAUT 1 (AGO1), which is part of the RNA-induced silencing complex (RISC). Guided by homology between the miRNA sequence and its target mRNA, RISC cleaves the mRNA using AGO1 resulting in downregulation of gene activity [2]. Mismatches (up to three) in the 3′ portion of the miRNA-target duplex can typically be tolerated, but cleavage efficiency is greatly reduced by the presence of mismatches near the 9th–12th nucleotide from the 5′ end of the miRNA-target duplex [6]. The binding of a miRNA with low cleavage efficiency can elicit translational inhibition of the target mRNA, which ultimately leads to downregulation of protein levels of the targeted gene [7, 8]. Plant miRNAs are known to be responsive to a range of stresses, including drought [9, 10], salinity [11, 12] and pathogen invasion [13, 14]. Mutualistic interactions also affect host miRNA expression, as seen following inoculation with mutualistic microbes such as diazotrophic bacteria [15, 16] and arbuscular mycorrhizae (AM) [5, 17, 18].

Arbuscular mycorrhizae are symbiotic root endophytic fungi that colonize the root systems of 70 to 90% of land plants [19]. They are generally considered mutualists that facilitate water and mineral transfer from the soil to the host in exchange for photosynthetically derived carbon from the host plant. AM structures have been documented in fossils that are around 450 million years old [20], in an era when most land plants had moss-like growth habits, and it has been hypothesized that AM were influential in the colonization of land by aquatic plants [20]. AM are intimately associated with plant roots. Hallmark structures called arbuscules are tree-like in appearance and serve as the sites of exchange between the plant and the fungus. The hyphae extend from the root cortical cells into the rhizosphere, often connecting neighboring plants [21]. The AM contribution of phosphorus (P) to plants has been demonstrated to be a major mode of plant P acquisition [22]. Even in P supplemented soils and in the absence of apparent growth increases, over 50% of ^32^P uptake was contributed by AM in wheat [22]. Symbiosis with AM fungi may be especially beneficial in low-input agricultural systems.

Evidence suggests that AM-responsive miRNAs regulate genes involved in an array of processes. Their presence in the phloem suggests that they also have the potential to act as AM-triggered long-range signaling molecules [5]. The miRNA171 family controls AM infection rates in model legumes [23–25]. This miRNA family targets GRAS transcription factors, some of which are responsible for coordinating transcription of components of the strigolactone biosynthetic pathway [26]. Auxin response factors have also been shown to be regulated by AM-responsive miRNAs, including miR160, a miRNA family that is highly conserved across land plants [5, 27]. While there are several miRNA families that are ubiquitous and highly conserved across plant species, many miRNAs are family- or species-specific [27]. Therefore, studying global miRNA activity in species of interest is essential to understand species-specific aspects of AM colonization.

Due to its ability to thrive on marginal land and produce large amounts of biomass, switchgrass (*Panicum virgatum*) is an attractive candidate feedstock for cellulosic biofuel production. There are two well-described ecotypes of switchgrass, upland and lowland, generally adapted to northern and southern US latitudes, respectively. Upland ecotypes are smaller and typically more tolerant to drought than lowland ecotypes [28, 29]. Both ecotypes are responsive to nitrogen (N) and phosphorus (P) fertilization, meaning there is potential to increase yield with soil amendments, especially when growing switchgrass on marginal lands [28–31]. Given their role in nutrient uptake and ability to confer abiotic stress tolerance to the host, AM fungi could help maximize switchgrass biomass production under low nutrient regimes. In this study, we focused on the identification of miRNAs responsive to AM colonization in switchgrass. We profiled genome-wide miRNA expression changes in Alamo (lowland), Dacotah (upland), and two upland-lowland F_1_ hybrids in AM-inoculated and control conditions. We used degradome sequencing to identify targets of the differentially expressed miRNAs to gain insights into the genetic pathways under their regulation. We identified 94 known miRNAs and a further 28 novel and potentially switchgrass-specific miRNAs. A common characteristic of AM-colonized switchgrass genotypes was the repression of miRNAs belonging to the “copper miRNA group” and we demonstrated that these miRNAs were targeting genes encoding blue copper proteins, superoxide dismutases, and laccases*.* Some switchgrass genotypes also showed upregulation of miR171 families and the related miR479 family, demonstrating the complexity of miRNA-mediated AM regulation*.*

## Results

### MicroRNA identification

MicroRNA libraries were generated for switchgrass lowland cultivar Alamo, upland cultivar Dacotah, and two F_1_ hybrids (F1–304H and F1–346L) derived from a lowland (Alamo genotype AP13) x upland (Summer genotype VS16) cross grown in the absence and presence of AM. The number of raw and filtered reads obtained for each library is presented in Supplemental Table S1 (Additional file 1). From the filtered read set, pooled by accession across treatments and replicates, miRDeep-P2 identified 182, 195, 219 and 225 miRNA precursors in the Alamo, Dacotah, F1–304H and F1–346L read pools, respectively (Supplemental Table S2, Additional file 1). MicroRNAs with an identical mature sequence but originating from precursors that mapped to different genomic loci, were considered paralogous. After removing redundancy in the miRNA precursor lists across the four read pools and collapsing paralogous mature miRNAs into one representative miRNA, we identified 141 distinct mature miRNA sequences. Fourteen isomiRs (mature miRNAs with slightly varying sequence but derived from the same precursor sequence) were detected among the mature set, generally varying by 1 bp (Supplemental Table S3, Additional file 1). MiRNA clusters were defined as genomic regions shorter than 5 kb that harbored multiple miRNA-producing genes. Ten miRNA clusters were identified (Supplemental Table S3, Additional file 1). All of the identified clusters encoded miRNAs of a single family specific to that cluster. The two largest clusters were for the miR395 family located on chromosomes 7 K and 7 N, and consisted of five and four miRNA genes, respectively. These clusters span 761 bp on chromosome 7 K and 667 bp on 7 N. The majority of the clusters contained two miRNA genes and ranged in size from 419 bp to 4765 bp. Five precursors produced more than one mature miRNA (Supplemental Table S3, Additional file 1).

Approximately half of the mature miRNAs (72 miRNAs) were transcribed from only a single gene or gene cluster. Of the remaining half, 37 mature miRNAs were transcribed from two homoeologous miRNA genes, and 32 were transcribed from at least two non-homoeologous miRNA genes. However, within the latter group, homoeologous genes were present for 19 mature miRNAs (Supplemental Table S4, Additional file 1). For example, genes that were the source of precursors for Pv8_miR167 were detected on four sets of homoeologous chromosomes and a further two non-homoeologous chromosomes, while genes for precursors of Pv9_miR160 were detected on five sets of homoeologous chromosomes (Supplemental Table S4, Additional file 1).

Using the 141 representative mature miRNAs as queries against miRBase release 22 (http://www.mirbase.org/) revealed that 94 of them belonged to 33 known miRNA families (Supplemental Table S3, Additional file 1). The 58 known mature miRNAs belonging to 28 families identified in Alamo originated from 153 precursors, the 57 known miRNAs belonging to 26 families in Dacotah were derived from 153 precursors, the 67 known miRNAs belonging to 30 conserved families in F1_304H were produced from 169 precursors, and the 70 known miRNAs belonging to 30 conserved families in F1_346L originated from 175 precursors (Supplemental Figure S1, Additional file 2; Supplemental Table S2, Additional file 1). The miRNA gene family with the most members was miR156, for which 22 expressed miRNA genes were identified in F1–346L (Supplemental Figure S1, Additional file 2). To enhance confidence in the novel miRNAs, we eliminated potential novel miRNAs that were predicted in only a single read pool, resulting in 28 (out of 47) high confidence novel miRNAs. Among the predicted set of miRNAs, the dominant length was 21 nucleotides (nt) for both novel and known miRNAs (Supplemental Figure S1, Additional file 2).

### MicroRNA expression changes following AM inoculation

The presence of AM fungi in AM-inoculated plants and their absence in mock-inoculated control plants was verified using both microscopy (Supplemental Figure S2, Additional file 3) and PCR with the AM fungi-specific primers FLR3/FLR4 [32] (Supplemental Figure S2, Additional file 3). Colonization levels in AM-inoculated Alamo and Dacotah plants averaged 60.3 and 56.0% of the total root length, respectively (Supplemental Figure S2, Additional file 3). Total AM colonization rates in F1–304H and F1–346L were 29.4 and 39.8%, respectively, with the presence of vesicles being significantly higher in F1–346L (20.4%) than in F1–304H (12.4%) (Supplemental Figure S2, Additional file 3). In both experiments, we observed a higher number of vesicles compared to arbuscules. This may indicate that the AMF extracted more cost than it provided benefits to switchgrass plants at the examined time points [33]. No AM specific amplification was observed in mock-inoculated plants (Supplemental Figure S2, Additional file 3).

**Fig. 1:**
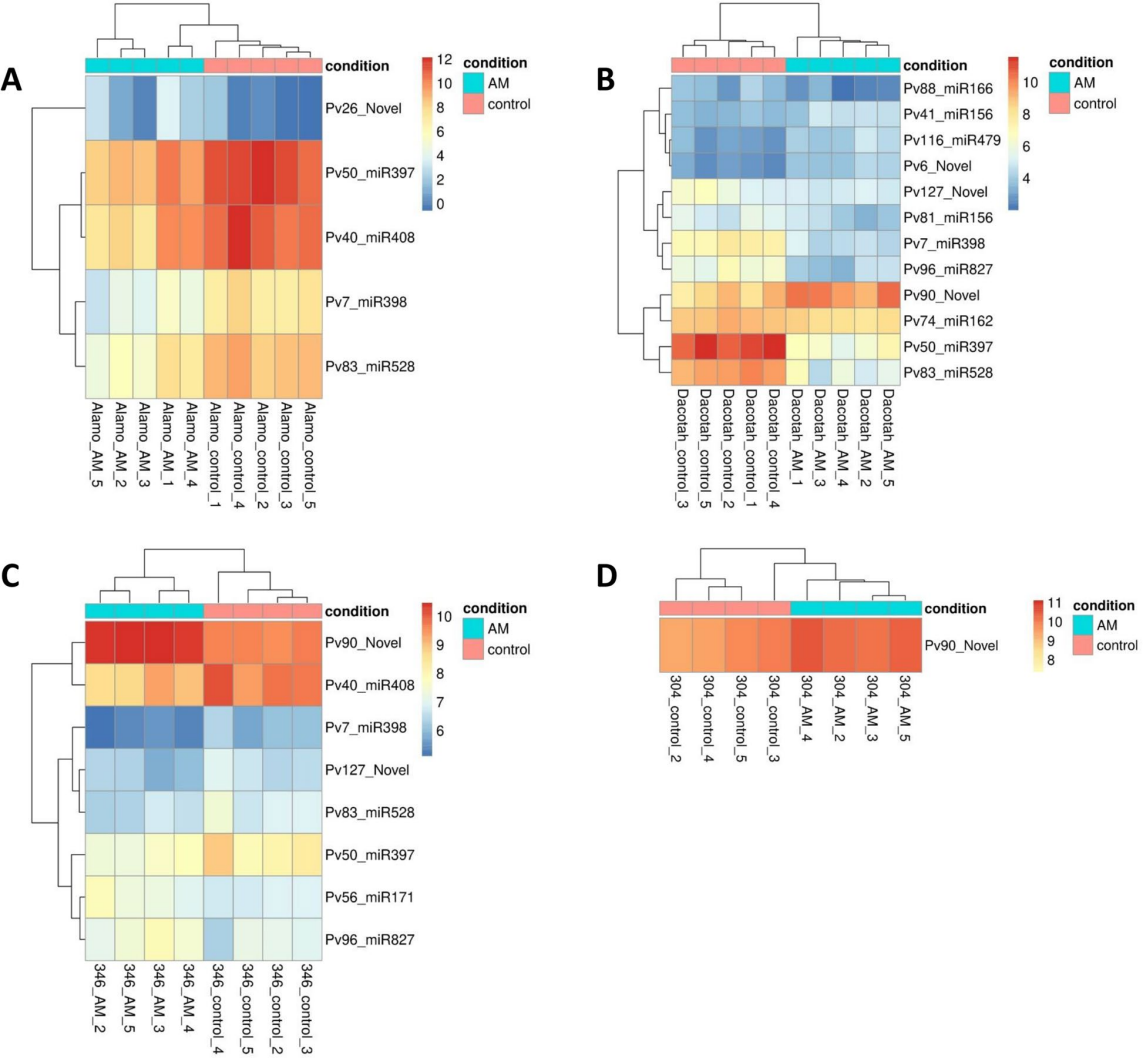
miRNA expression in AM vs control samples in Alamo (**A**), Dacotah (**B**), F1–346L (**C**) and F1–304H (**D**). Regularized log transformation was performed on the DEseq2 output values prior to generating the heatmaps using rlogTransformation() in R version 4.1.2. miRNAs that were differentially expressed between AM and control samples at padj < 0.05 are shown

To quantify miRNA expression in these plants, read counts against known and novel mature miRNAs were used to determine their relative abundance. We profiled expression of the 94 known and 28 novel mature miRNAs annotated from the four library pools (a library pool corresponds to all treatments and replicates for a single accession). In the Alamo/Dacotah experiment (Fig. 1; Supplemental Table S5, Additional file 1), five and 12 miRNAs, four of which were common between the two accessions, were differentially expressed (DE) in AM- compared to mock-inoculated plants in Alamo and Dacotah, respectively. The majority of DE miRNAs were downregulated in AM-inoculated plants compared to control plants (Fig. 1; Supplemental Table S5, Additional file 1). In the F1–304H/F1–346L experiment (Fig. 1; Supplemental Table S5, Additional file 1), F1–304H had a single differentially expressed miRNA (Pv90_Novel). This miRNA was also upregulated in F1–346L and Dacotah (Fig. 1; Supplemental Table S5, Additional file 1). F1–346L had an additional seven differentially expressed miRNAs, five of which were downregulated in AM-colonized plants.

**Fig. 2:**
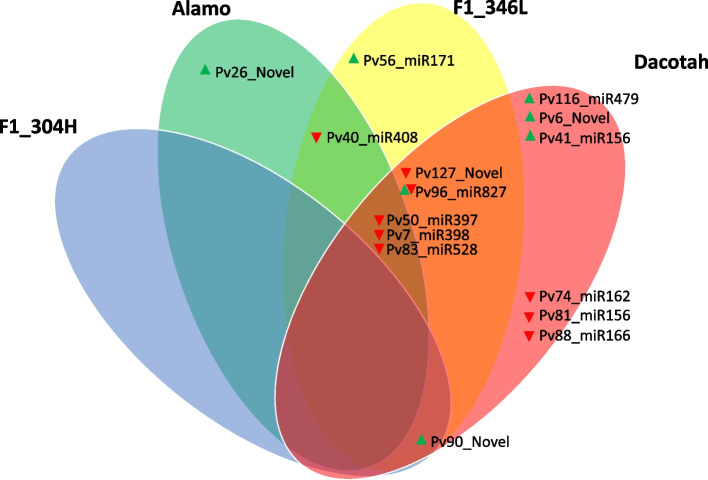
Differentially expressed (DE) miRNAs across accessions. Arrows show the direction of expression change due to AM colonization; red arrow down = downregulated, green arrow up = upregulated. MiRNAs were considered DE if padj< 0.05 when comparing AM vs. control. Pv96_miR827 has two opposing arrows as it is upregulated in F1–346L and downregulated in Dacotah

Comparing the differentially expressed miRNAs across the two experiments (four accessions) revealed four miRNAs, Pv90_Novel, Pv50_miR397, Pv7_miR398, and Pv83_miR528, that were DE in the same direction across three of the four genotypes (Fig. 2). Three additional miRNAs, Pv40_miR408, Pv127_Novel, and Pv96_miR827 were differentially expressed between AM-inoculated plants and controls in two genotypes. MicroRNAs that were DE in more than one genotype were considered part of a core response of switchgrass to AM colonization. We considered the rest of the miRNAs to be potentially part of a genotype-dependent response to AM.

### MicroRNA target analysis

**Fig. 3:**
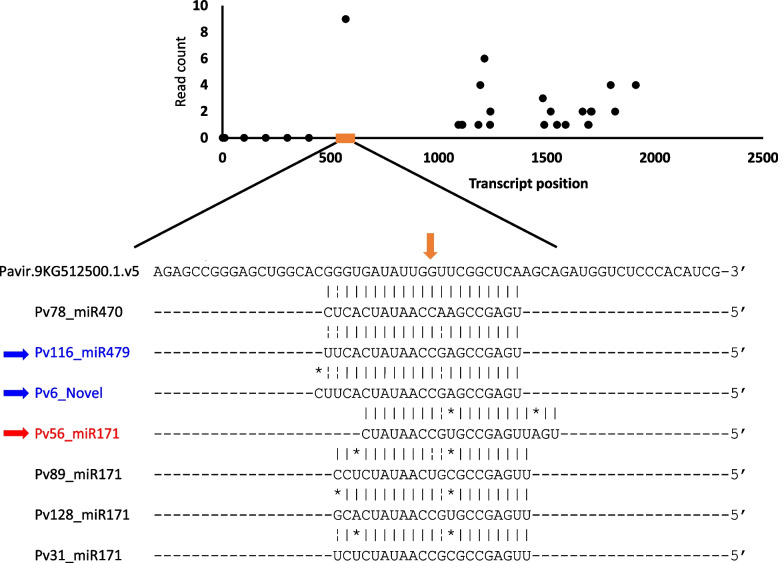
Target site analysis of *NSP2*. Each data point in the plot represents the 5′ position of a degradome read mapped to the reference transcriptome. The orange box indicates the position of the potential recognition site (transcript nucleotide position 570). The enlarged window is an alignment of the miRNAs with homology to this recognition site with the orange arrow representing the cleavage position, blue arrows pointing to miRNAs that were found to be AM responsive in Dacotah, and the red arrow pointing to an AM-responsive miRNA in F1–346L. “|” indicates Watson-Crick base pairing, “¦” indicates G-U wobble, and “*” indicates a mismatch with the Pavir.9KG512500.1. target site

We used degradome sequencing to identify target genes and gain insights into the genetic pathways targeted by the DE miRNAs detected in the expression profiling experiments. We identified strongly supported degradome signatures for 38 miRNA-target gene pairs representing 10 of the 15 DE miRNAs (Table 1). The gene families most frequently targeted by the miRNAs were laccases, GRAS transcription factors and blue copper proteins (Table 1; Supplemental Table S6, Additional file 1). Target discovery was skewed towards known miRNAs, with 89.1% of the targets being regulated by known miRNAs and 10.9% being controlled by novel miRNAs. For several upregulated and downregulated miRNAs during AM colonization, homoeologous K and N target genes were identified with strong degradome signatures (Table 1; Supplemental Table S6, Additional file 1). Several miRNAs also regulated multiple members of the same gene family. For example, *LAC3*, *LAC10*, *LAC13*, *LAC17* and *LAC23* all had strong degradome signatures for Pv50_miR397 (Supplemental Table S6, Additional file 1). Similarly, multiple GRAS family transcription factors were targeted by both known and novel miRNAs (Table 1; Supplemental Table S6, Additional file 1). A GRAS family transcription factor that has previously been shown to be relevant to AM colonization, *Nodulation signaling pathway 2 (NSP2)* [23, 24], had cleavage signatures from two switchgrass miRNAs that were upregulated by AM colonization, Pv116_miR479, and Pv6_Novel (Table 1; Supplemental Table S6, Additional file 1). Pv116_miR479 had a higher minimum free energy (MFE) ratio than Pv6_Novel at 0.96 and 0.89, respectively (Supplemental Table S6, Additional file 1). This difference is likely due to the difference in length between the two miRNAs, with the extra base in Pv6_Novel being mismatched at the 5′ end of the miRNA and target site alignment (Fig. 3). A common feature of these two miRNAs that targeted *NSP2* and were responsive to AM in Dacotah was that they both had base-pairing at the cleavage position, a G-U wobble one position 3′ of the cleavage base (nucleotide position 570), and matching bases continuing toward the 3′ region of the recognition site (Fig. 3). Surprisingly, Pv78_miR479 had perfect complementarity to the target site except for the penultimate (5′) base but was not found to be AM-responsive. The mean number of normalized counts across all samples in Dacotah was similar for Pv78_miR479, Pv116_miR479 and Pv6_Novel at 14.01, 16.09, and 13.55, respectively (Supplemental Table S7, Additional file 1). Given the similarities in the abundance of these three miRNAs, it is difficult to pinpoint which one is responsible for cleaving *NSP2*. Possibly, all three miRNAs cleave *NSP2*.

**Table 1:** Degradome-verified targets of AM-responsive miRNAs^c^

miRNA	Gene	PFAM Description^a^	Best BLASTP hit to NCBI SwissProt - Viridiplantae (E-value)^b^
Pv40_miR408	Pavir.6NG279000	Cu_bind_like	Blue copper protein – *Pisum sativum* (2e-33)
Pv40_miR408	Pavir.6KG319400	Cu_bind_like	Blue copper protein – *Pisum sativum* (2e-33)
Pv83_miR528	Pavir.2KG499500	Cu_bind_like	Mavicyanin – *Cucurbita pepo* (6e-36)
Pv83_miR528	Pavir.2NG547000	Cu_bind_like	Mavicyanin – *Cucurbita pepo* (6e-36)
Pv50_miR397	Pavir.5KG613400	Cu-oxidase_2	Laccase-3 – *Oryza sativa* (0.0)
Pv40_miR408	Pavir.1NG406200	Cu_bind_like	Basic blue protein – *Arabidopsis thaliana* (1e-38)
Pv50_miR397	Pavir.5NG569300	Cu-oxidase_2	Laccase-3 – *Oryza sativa* (0.0)
Pv7_miR398	Pavir.9NG593500	Sod_Cu	Superoxide dismutase [Cu-Zn] 4A – *Zea mays* (5e-100)
Pv40_miR408	Pavir.4NG301700	Cu_bind_like	Blue copper protein – *Pisum sativum* (1e-41)
Pv7_miR398	Pavir.3KG207096	COX5B	Cytochrome c oxidase subunit 5b-2, mitochondrial – *Arabidopsis thaliana* (2e-53)
Pv96_miR827	Pavir.7NG296900	SPX	SPX domain-containing membrane protein OsI_17046 – *Oryza sativa* (4e-114)
Pv50_miR397	Pavir.9NG700300	Cu-oxidase_2	Laccase-10 – *Oryza sativa* (0.0)
Pv50_miR397	Pavir.5NG584800	Cu-oxidase_2	Laccase-12 – *Oryza sativa* (0.0)
Pv50_miR397	Pavir.7KG245300	Iso_dh	Isocitrate dehydrogenase [NADP], chloroplastic/mitochondrial – *Arabidopsis thaliana* (0.0)
Pv50_miR397	Pavir.8NG004003	Cu-oxidase_2	Laccase-17 – *Oryza sativa* (0.0)
Pv50_miR397	Pavir.8KG177620	Cu-oxidase_2	Laccase-23 – *Oryza sativa* (0.0)
Pv83_miR528	Pavir.4KG213105	Cu_bind_like	Blue copper protein – *Pisum sativum* (3e-09)
Pv83_miR528	Pavir.4NG131900	Cu_bind_like	Blue copper protein – *Pisum sativum* (8e-10)
Pv50_miR397	Pavir.9KG330400	Ribosomal_L18p	–
Pv41_miR156	Pavir.1NG540200	Dev_Cell_Death	DCD domain-containing protein NRP – *Arabidopsis thaliana* (8e-17)
Pv41_miR156	Pavir.1KG509700	Dev_Cell_Death	DCD domain-containing protein NRP – *Arabidopsis thaliana* (3e-16)
Pv7_miR398	Pavir.7NG297300	HMA	Copper chaperone for superoxide dismutase, chloroplastic – *Oryza sativa* (1e-154)
Pv26_Novel	Pavir.4KG252700	#N/A	–
Pv6_Novel	Pavir.9KG512500	GRAS	Protein NODULATION SIGNALING PATHWAY 2 *– Oryza sativa* (0.0)
Pv56_miR171	Pavir.1NG390019	GRAS	Scarecrow-like protein 6 – *Arabidopsis thaliana* (2e-91)
Pv56_miR171	Pavir.7KG283500	GRAS	Scarecrow-like protein 6 – *Arabidopsis thaliana* (1e-92)
Pv116_miR479	Pavir.9KG512500	GRAS	Protein NODULATION SIGNALING PATHWAY 2 *– Oryza sativa* (0.0)
Pv116_miR479	Pavir.9NG665600	GRAS	Protein NODULATION SIGNALING PATHWAY 2 *– Oryza sativa* (0.0)
Pv56_miR171	Pavir.4NG003100	GRAS	Scarecrow-like protein 6 – *Arabidopsis thaliana* (2e-82)
Pv6_Novel	Pavir.9NG665600	GRAS	Protein NODULATION SIGNALING PATHWAY 2 *– Oryza sativa* (0.0)
Pv56_miR171	Pavir.7NG276500	GRAS	Scarecrow-like protein 6 – *Arabidopsis thaliana* (2e-95)
Pv56_miR171	Pavir.1KG431100	GRAS	Scarecrow-like protein 6 – *Arabidopsis thaliana* (2e-92)
Pv26_Novel	Pavir.3KG081543	#N/A	–
Pv26_Novel	Pavir.8KG229500	Dirigent	Pterocapan synthase – *Glycine max* (2e-23)
Pv26_Novel	Pavir.5KG344400	DUF3511	–
Pv40_miR408	Pavir.9NG638900	FAE1_CUT1_RppA	3-ketoacyl-CoA synthase 6 – *Arabidopsis thaliana* (0.0)
Pv40_miR408	Pavir.3KG377542	#N/A	–
Pv50_miR397	Pavir.1KG421500	MIP	Aquaporin TIP2–2 – *Zea mays* (3e-163)

^a ^PFAM descriptions obtained from https://phytozome-next.jgi.doe.gov/phytozome/

^b ^indicates no homology at E-value <1e-5

^c ^Additional degradome information can be found in Supplemental Table S5, Additional file 1

## Discussion

### miRNA identification

Although computational and empirical efforts have previously been undertaken to catalogue miRNAs in switchgrass [34–36], at the time of our research, no annotated switchgrass miRNAs were present in miRbase (version 22). Furthermore, while previous genome-wide miRNA studies in switchgrass focused on miRNAs expressed under abiotic stress [35, 36], and in emerging tillers and inflorescences [37], ours is the first study to investigate miRNA involvement in microbial interactions with switchgrass roots. We detected between 26 and 30 miRNA families in each of our four library pools generated from roots from switchgrass plants 70–84 days after mock- and AM-inoculation (Supplemental Table S2, Additional file 1). The number of miRNA families discovered in each accession in our study is similar to the numbers previously reported in other switchgrass studies that focused on miRNAs. Using public sequence databases and comparative genomics methods, Xie et al. [34] detected 121 putative miRNA genes belonging to 44 miRNA families. The use of next-generation sequencing led to the identification of 29 and 28 known miRNA families in leaves from 80-day old plants [35] and in emerging tillers and inflorescences [37], respectively. A total of 670 miRNA families was reported by Xie et al. in 10-day old seedlings. As our reported miRNA numbers only include miRNAs with identified precursors, we calculated the number of unique miRNA families from Xie et al. (54 families) from their supplementary data for comparison. The most numerous miRNA gene family reported by Xie et al. was miR395 with 13 members, followed by miR156 and miR166 with nine and seven members, respectively. We also identified 13 members for miR395, 17–20 (depending on the plant genotype analyzed) for miR156, and 14 for miR166. Members of these miRNA gene families are also highly represented in other monocot miRNA studies. For example, in wheat, miR166 was found to have ~ 24 members [38] while in sorghum, a diploid monocot, miR166 with seven members was one of the largest families identified in anthers [39]. Our study is the most recent effort to identify expressed miRNA loci in switchgrass, and in combination with previous reports, provides the switchgrass research community access to miRNA annotations that span different tissue types and developmental stages. Additionally, we identified miRNAs expressed in the upland and lowland accessions Dacotah and Alamo, respectively, and in two F_1_ hybrids from a lowland x upland cross (Alamo X Summer). MiRNA expression was largely similar across these accessions in terms of the number of miRNAs discovered (Supplemental Table S2, Additional file 1; Supplemental Figure S1, Additional file 2). Some variation seen in miRNA family sizes may result from similar mature sequences being expressed from different loci in the different genetic backgrounds. We may also have missed miRNA loci that showed sequence variation between the different accessions. At the time of this study, the most robust switchgrass reference genome sequence was of AP13 (lowland) in its 5th version [40] (https://phytozome-next.jgi.doe.gov/info/Pvirgatum_v5_1). The discovery of high confidence upland-specific novel miRNAs will be facilitated as genome assemblies of Dacotah, Summer and other uplands become available.

### Copper-miRNAs are downregulated in response to AM colonization

A total of 15 miRNAs were identified across the four plant genotypes tested that were responsive to AM inoculation. We identified target mRNAs through degradome sequencing for ten of these miRNAs (Table 1; Supplemental Table S6, Additional file 1). Four of the most significantly DE known miRNAs in our analyses, Pv7_miR398, Pv50_miR397, Pv83_miR528, and Pv40_miR408 (Fig. 1; Supplemental Table S5, Additional file 1), are part of a group called the copper (Cu) miRNAs which are upregulated under copper limitation [41–43]. They cleave non-essential transcripts encoding proteins that utilize copper, thus liberating the copper for use as a cofactor by more essential proteins [41]. In our study, all Cu-miRNAs were downregulated in the presence of AM. It seems unlikely that the downregulation of the Cu-miRNAs in our study was due to a Cu-deficiency in the control versus AM-inoculated plants. While AM fungi can deliver copper to plants [44] and, hence, could alleviate Cu deficiency, our fertilizer solution contained copper, and neither the control nor the AM-inoculated plants displayed symptoms of copper deficiency. However, members of the Cu-miRNA group have also been shown to have altered expression patterns under other abiotic stress conditions, including drought [10], and following microbial interactions [15, 45, 46]. It has been reported that Cu-miRNAs are downregulated in the host following pathogenic interactions and induced upon colonization with mutualists [41]. However, other studies conflict with this view [18, 45], suggesting that up- or downregulation of Cu-miRNAs following biotic interactions may depend on the microbe-host system.

Pv7_miR398 was downregulated in AM-inoculated compared to control (mock-inoculated) plants in Alamo, Dacotah, and F1–346L (Fig. 1; Supplemental Table S5, Additional file 1). Degradome signatures for Pv7_miR398 were found for transcripts encoding superoxide dismutase (SOD) (Supplemental Table S6, Additional file 1). MiR398 also targets *SOD* transcripts in *Arabidopsis* [47]. SOD mitigates the effects of oxidative stress by detoxifying superoxide radicals and converting them into the less harmful hydrogen peroxide (H_2_O_2_). Antioxidant enzyme activity (including SOD activity) has been shown to increase in response to mycorrhizal colonization in a range of plant species, including lettuce*,* wheat, tomato and maize [38, 48–51]. These studies demonstrated that AM colonization can increase antioxidant enzyme activity, but a mechanism for the response of antioxidant gene expression to AM colonization involving miRNA had not yet been established. We provide evidence that Pv7_miR398 is downregulated in switchgrass in response to AM colonization and that it is involved in the cleavage of *SOD* transcripts, suggesting that *SOD* regulation upon AM colonization is miRNA controlled. Downregulation of miR398 and concomitant upregulation of SOD has similarly been seen upon infection with pathogens as well as during early stages of colonization by mutualistic diazotrophic bacteria [45, 52–54]. It is likely that the establishment of a symbiotic relationship activates a broad-spectrum defense response in the host [55] which beneficial AM fungi somehow avoid or overcome [56]. Our results support this observation by providing a link between AM colonization and SOD activity through the downregulation of miR398. Despite SOD activity being correlated with the inhibition of fungal pathogens, we know that AM fungal growth was not deeply inhibited, because fungal structures were present in the AM inoculated plants (Supplemental Figure S2, Additional file 3). Precise manipulation of antioxidant level, timing, or location by plant tissues may be a way to limit colonization to host-preferred fungi but the basis of this differentiation between desired and undesired fungi is not known. Nonetheless, our data suggest that this process is under miRNA control.

Another Cu-miRNA that was downregulated in roots of AM- compared to mock-inoculated switchgrass plants was miR397. Among the documented targets of miR397 are *laccase* genes [57, 58]. We identified transcripts from five laccase genes with degradome signatures from Pv50_miR397. Laccases are multicopper oxidases that are involved in a multitude of processes [59]. Although the functions of the laccases that were regulated by miR397 in our study are unknown, we focus here on well-studied involvement of laccases in lignin deposition because this is of particular interest for switchgrass from a bioenergy point of view [57, 60]. Overexpression of miR397 in *Arabidopsis* and *Populus* resulted in reduced lignin [58, 61]. Furthermore, root colonization by microbial communities in barley was positively correlated with root and shoot lignin content [62]. We therefore hypothesize that switchgrass plants respond to AM colonization by increasing lignin content through the suppression of Pv50_miR397 and subsequent upregulation of laccase genes. Verification of this hypothesis will require measurement of root and/or shoot lignin content. Of course, other models can also be proposed that involve roles of laccases in defense, and/or polymerization of phenolic compounds, for instance [59], as part of the interaction with AMF.

The remaining two members of the copper miRNA group that were downregulated by AM colonization in our study are Pv83_miR528 and Pv40_miR408. MiR408 has been shown to also be downregulated in tomato plants inoculated with AM [63]. Pv83_miR528 targeted two sets of homoeologous transcripts that encode the various electron transfer agents called blue copper proteins (BCPs), including mavicyanin (Table 1). Pv40_miR408 also targeted genes encoding BCPs in all degradome libraries analyzed in our study (Supplemental Table S6, Additional file 1). Upregulation of BCPs, including mavicyanin and the BCPs *MtBcp1a* and *MtBcp1b*, has previously been observed in AM colonized roots of *Helianthus* and *Medicago truncatula*, respectively [64–66]. In *M. truncatula*, expression of *MtBcp1a* and *MtBcp1b* was highly correlated with AM colonization level and the amount of arbuscules, suggesting that BCPs play a role in symbiotic function [65, 66]. Collectively, the results suggest that AM colonization downregulates both miR528 and miR408 which, in turn, leads to upregulation of specific sets of genes encoding blue copper proteins.

### MiRNAs controlling specific GRAS transcription factors are upregulated in response to AM

The most highly upregulated miRNAs in AM colonized roots found in our study with a strong degradome signature were Pv116_miR479 and Pv6_Novel, which cleave *Nodulation signaling pathway 2 (NSP2)* transcripts. *NSP2* codes for a GRAS family transcription factor that promotes biosynthesis of strigolactone, a plant hormone that plays an essential role in host-AM fungal interactions. *NSP2* cleavage by miRNAs in response to AM colonization has been well described in *M. truncatula* [23, 24]. In *Medicago*, *NSP2* was suppressed by miR171, which limited AM infection throughout the root system. MiR171 expression is dependent on nutrient availability and is upregulated under high nutrient availability, hence restricting AM colonization [24]. We observed Pv116_miR479 upregulation in both Alamo and Dacotah under AM vs. control conditions, although the expression difference was not statistically significant in Alamo (Supplemental Table S7, Additional file 1). MicroRNA cleavage efficiency is dependent on matching bases near the middle of the target site [6]. We detected a cleavage signature for *NSP2* at position 570 (Fig. 3). Pv78_miR479 was perfectly matched to this position but was not AM responsive. The AM-responsive miRNA Pv116_miR479, on the other hand, had a G-U wobble one base 5′ to the cleavage position. This G-U wobble was present in several other miR171 family members (Fig. 3). Some miR171 family members have been shown to enhance AM colonization by blocking access to and thereby preventing degradation of their targets by miRNAs with higher levels of homology [25]. Given the similarity in the abundance of miRNA Pv116_miR479 and Pv78_miR479, it is possible that *NSP2* cleavage is directed by Pv78_miR479 and that the AM-responsive Pv116_miR479 with mismatches near the target site promotes AM colonization by blocking *NSP2* degradation in certain cells. Future cell-type-specific expression studies are needed to shed light on this mechanism.

Pv56_miR171 was upregulated only in F1–346L plants. This miRNA directed cleavage of transcripts of scarecrow-like (SCL) proteins (Table 1), which are GRAS family transcription factors. In tomato, several *SCL* genes were upregulated in cells containing arbuscules [67]. RNAi silencing of those genes delayed AM development, marked by fewer arbuscules [67]. Other root symbioses are also controlled by *SCL*s. In soybean, *SCL6* is involved in nodulation and is under the control of miR171. Overexpression of miR171 leads to a reduction in nodulation [68]. Interestingly, in our study, Pv56_miR171 was upregulated under AM colonization, presumably leading to a downregulation of *SCL* genes. Extrapolating from the observation in other studies, this would lead to a reduction in AM colonization. Pv56_miR171 was upregulated only in F1–346L, a line that was originally selected because of low AM colonization rates in the field, although this line did not show reduced AM colonization in our greenhouse experiments. However, this genotype harbored more vesicles than F1–304H (Supplemental Figure S2, Additional file 3). It is possible that the AM strains that colonized the switchgrass plants in the field were different from those used in our greenhouse experiments. Still, it will be interesting to knock out the set of *SCL* genes controlled by Pv56_miR171 and observe the effect on AM colonization.

## Conclusion

We identified AM-responsive miRNAs in upland and lowland switchgrass accessions, as well as upland-lowland F_1_ hybrids. Cu-miRNA downregulation was a feature common to three of the four genotypes tested. Their targets, *SOD* and laccase mRNAs, suggest that the Cu-miRNAs are controlling defense pathways leading to ROS detoxification and, potentially, lignin deposition in switchgrass, similar to what has been shown in annual crops and model species. An effect of AM colonization on lignin would be pertinent to switchgrass optimization for biofuels and needs to be further investigated. Also, further studies will need to clarify whether the response seen here to AM fungi differs from a response to pathogenic infections in switchgrass and whether this response varies during the establishment, maintenance and degeneration of the host-AMF symbiosis. Given the high abundance of vesicles in our analysis, it seems like that these samples were mostly in the degeneration stage.

MiRNAs that target genes encoding BCPs were also downregulated. While upregulation of BCPs has been shown in several studies to be associated with higher levels of arbuscules, the precise role of these proteins in AM colonization remains unknown. Our study also demonstrated the complexity of GRAS transcription factor regulation by AM-responsive miRNAs. Several GRAS transcription factors that are typically upregulated in AM colonized roots were shown to be under miRNA control in our study. However, contrary to the expectation, the expression of those miRNAs was upregulated, which would lead to a downregulation of the target genes. The identification of closely related miRNAs, some of which were perfectly matched at the critical bases, while others had one or two mismatched bases at the cleavage site, indicates that target gene levels may be determined by differential ratios of miRNAs with blocking capacity and miRNAs with cleavage capacity.

In summary, this study demonstrated the role that miRNAs play in colonization of switchgrass by AM fungi. While several miRNA targets had previously been shown to be differentially regulated under AM colonization in other species, our study shows that several of those genes are under the control of miRNAs in switchgrass. Considering the conserved nature of those miRNAs, this likely holds true for other species.

## Methods

### Plant material and growth conditions

Seeds of *Panicum virgatum* accession “Dacotah” (origin South Dakota, lot KN04272, product PVDA) and accession “Alamo” (origin Texas, lot YE01165, product PVAL) were obtained from Applewood Seed Company. Seeds were surface sterilized with 1% NaOCl for 5 min, rinsed three times with sterile double deionized (dd) H_2_O, and germinated in petri plates on moist filter paper. At the two-leaf stage, seedlings were transplanted to Ray Leach cone-tainers (164 mL) containing an autoclaved mixture of sand:turface:vermiculite 2:2:1 v/v/v.

Two F_1_ progeny, F1–304H and F1–346L, from a cross between the lowland genotype AP13 and upland genotype VS16 [69] were selected in a field-based study as high and low AM colonizers, respectively. The F_1_ progeny were propagated from greenhouse-maintained tillers using nodal culture [70] to generate AM-free clonal replicates. Leaf sheaths were peeled back from tillers to reveal the axillary bud. If no axillary bud was observed, the node was discarded. The tiller was cut 2 cm below the node and 5 cm above the node to yield an approximately 8 cm stem fragment. The stem fragments were surface sterilized with 1% NaOCl for 3 min and rinsed three times in filter-sterilized (0.22 μm) ddH2O. Segments were planted into autoclaved fine vermiculite saturated with 0.25x Murashige and Skoog Basal Salt Mixture (MS; Sigma- Aldrich) so that the axillary buds were about 1 cm below the surface. The node-containing stem segments were placed in a growth chamber under a 16 hr. day length and a constant temperature of 30 °C. When the shoots that emerged from the nodes were at the two-leaf stage, plants were moved to autoclaved vermiculite saturated with rooting solution (0.5x MS with 5 mg/L 1- naphthaleneacetic acid (Sigma-Aldrich)). After roots were formed, the plants were planted in 5x5x30 cm pots in a mixture of sand:turface:vermiculite 2:2:1 v/v/v.

During transplanting, five biological replicates each of Alamo and Dacotah, and 10 replicates of F1–304H and F1–346L were inoculated with 15 g of live AM whole inoculum consisting of equal volumes of *Rhizophagus intraradices* (INVAM FL208), *Funneliformis mosseae* (provided by Richard Lankau, University of Wisconsin, Madison), and *Paraglomus occultum* (INVAM IA702). The same number of replicates for each genotype was inoculated with autoclaved AM whole inoculum (control). Cultures of the three AM species were maintained on sorghum plants in the UGA Plant Biology greenhouse for 4 months prior to harvest. Whole inoculum consisted of the entire sorghum-AM fungi root mass and all soil in the pot, which was homogenized using hand tools and stored in paper bags at 4 °C prior to mixing and inoculating the switchgrass plants. The switchgrass plants were grown in a growth chamber with a 16 hr. day length and 24 °C day/night temperatures. The experiments on Alamo and Dacotah, and on the two F_1_ progeny, were temporally spaced. Plants were watered daily with deionized (DI) water. The Alamo/Dacotah experiment was fertilized weekly with a 0.5x strength Hoagland’s solution [71], while the F1–304H/F1–346L experiment was fertilized once a week with a low phosphate (0.1 mM NH_4_H_2_PO_4_) 0.5x Hoagland’s solution for the first 5 weeks and once at 8 weeks. Roots were harvested 10 weeks (Alamo/Dacotah) or 12 weeks (F1–304H/F1–346L) post inoculation. Root tissue was rinsed free of soil, patted dry with a paper towel, flash frozen in liquid nitrogen and stored at − 80 °C prior to RNA extraction. A separate sample of root tissue from the F1–304H/F1–346L plants was stored in 50% ethanol at 4 °C for microscopic examination of AM structures. Since the entire root systems were sampled for RNA extraction, separate temporal replicates of Alamo and Dacotah seedlings grown under the same conditions were sampled to quantify AM structures using microscopy. Roots from three replicates of AM inoculated Alamo and Dacotah plants and controls were cut 2 cm from the crown, rinsed with tap water, and stored in 50% ethanol prior to staining.

### RNA extraction and small RNA library preparation

Root samples were ground under liquid nitrogen in a mortar and pestle. RNA from Alamo and Dacotah root samples was extracted using Trizol (Ambion) according to the manufacturer’s instructions with one additional chloroform extraction step and one additional 75% ethanol wash of the RNA pellet. RNA was treated with TURBO DNA-free DNAse (Invitrogen) to remove genomic DNA. RNA from F1–304H and F1–346L root samples was extracted using Trizol (Ambion) followed by purification and concentration on an RNA Clean & Concentrator^TM^-5 (Zymo Research) column according to the manufacturer’s instructions. Samples from two replicate plants were pooled before purification. DNaseI treatment was performed using the “column protocol” provided with the RNA Clean & Concentrator^TM^-5 (Zymo Research) kit, and the DNA-free total RNA was eluted with 15 μL nuclease-free water. RNA integrity was checked on a 1% agarose gel in 1x Tris/Borate/EDTA (TBE) or on a Bioanalyzer (Agilent) RNA 6000 nano chip.

Small RNA libraries were constructed from 400 ng of total RNA for each of the five replicates of each accession/treatment group in the Alamo/Dacotah experiment resulting in 20 barcoded libraries using the NEBNext® Small RNA Library Prep Set for Illumina® according to the manufacturer’s instructions. The cDNA libraries were PCR amplified for 14 cycles using barcoded primers. Verification that the final library comprised fragments in the size range 140–145 bp was assessed using a Fragment Analyzer™ Automated CE System at the Georgia Genomics and Bioinformatics Core (GGBC). The library DNA was diluted to 10 nM and libraries were sequenced on an Illumina NextSeq platform with single-end 75 bp read length at GGBC. Libraries for four replicates per genotype and treatment were similarly prepared for the F1–304H and F1–346L samples except that 800 ng of total RNA was used as input and 12 cycles of PCR were used for library amplification.

### Degradome library preparation

The library procedure as described by Willmann et al. [72] with some modifications was used to generate degradome libraries. Briefly, 400 ng of total RNA was pooled across the five replicates of Alamo and Dacotah resulting in one pool per treatment per accession containing 2 μg of total RNA. Ten micrograms of total RNA for two biological replicates of F1–304H and F1–346L from AM and control samples were used as input for degradome library preparation. Poly(A) selection steps were performed using the NEBNext® Poly(A) mRNA Magnetic Isolation Module according to the manufacturer’s instructions. To ligate the 5′ adapter to the uncapped poly(A) RNA, 10 μL of NEBNext® 3′ ligation buffer (2x), 1 μL of 11.25 μM NEBNext® 5′ adapter (denatured by incubating at 70 °C for 2 minutes and snap cooled on ice), 1 μL of NEBNext® 5′ ligation buffer (10x), and 2.5 μL of NEBNext® 5′ ligation mix were added to 15.5 μL poly(A) RNA. The ligation was performed at 25 °C in a thermocycler for 1 hr., after which a second poly(A) selection was carried out. Adapter-ligated RNA was eluted with 7.74 μL of nuclease free H_2_O to which 1.26 μL of a 20 μM custom NEBNext® 3′ adapter with a random hexamer 3′ tail (5′-AGACGTGTGCTCTTCCGATCTnnnnnn-3′) was added. Samples were incubated at 65 °C for 5 min followed by cooling at 4 °C for 2 min. Reverse transcription (RT) was done by adding 8 μL of NEBNext® First Strand Synthesis Reaction Buffer (5x), 1 μL of Murine RNase inhibitor, 1 μL of ProtoScript II Reverse Transcriptase (200 U/μl) and 10 μL of RNAse free H_2_O, and incubating the samples at 50 °C for 1 hr. followed by 15 min at 70 °C to deactivate the reverse transcriptase. The libraries were amplified using the following PCR conditions: 50 μL LongAMP 2x master mix, 2.5 μL of NEBNext® SR primer for Illumina, 2.5 μL of 10 μM NEBNext® indexing primer, 5 μL of nuclease-free H_2_O, and 40 μL of the RT reaction. The PCR program was 94 °C for 30 sec followed by 20 cycles of 94 °C for 15 sec, 62 °C for 30 sec, and 70 °C for 15 sec, followed by a final extension step of 70 °C for 5 min and 4 °C hold. For the F1–304H and F1–346L degradome libraries, the number of PCR cycles was reduced to 18. The PCR products of each library were purified individually using the QIAquick PCR Purification Kit according to the manufacturer’s instructions and fragments in the range 135–500 bp were selected on a 6% polyacrylamide gel. The library DNA was diluted to 10 nM and libraries were sequenced on an Illumina NextSeq platform with single-end 75 bp read length at GGBC.

### AM quantification using microscopy

The root material stored in 50% ethanol after harvest was prepared for microscopy by rinsing the roots with tap water and clearing them in 10% KOH at 90 °C for 15 to 20 min. After three to five rinses with ddH_2_O, the roots were acidified and stained. Alamo and Dacotah roots were acidified in 1% HCl for 80 min, stained with 0.05% trypan blue (500 mL glycerol, 0.5 g trypan blue crystals, 450 mL ddH_2_O and 50 mL 1% HCl) at 90 °C for 7 minutes, and destained in ddH_2_O for 1 hour at room temperature. F1–304H and F1–346L roots were acidified in 1% HCl for 1 hour at room temperature, stained with a 5% solution of Parker blue-black QUINK (http://www.parkerpen.com/en-US/ink-bottles) in 5% acetic acid at 90 °C for 15 min, rinsed three times in ddH_2_O and destained overnight in 5% acetic acid at room temperature. Roots were mounted longitudinally on glass microscope slides using polyvinyl-lacto-glycerol (PVLG) and AM quantification was performed as described in McGonigle and colleagues [32]. Microscopic quantification data were analyzed using the t.test function in R version 3.4.0.

### DNA extraction and AM quantification by PCR

A CTAB DNA extraction procedure based on Doyle and Doyle [73] was used to purify DNA from the frozen root tissue with the following modifications: The modified CTAB extraction buffer contained 1% 2-mercaptoethanol and 2% polyvinylpyrrolidone (f.w = 40,000). The samples were extracted once with an equal volume phenol:chloroform:isoamyl alcohol (25:24:1) and twice with chloroform:isoamyl alcohol (24:1). The DNA was precipitated by adding 0.1 volumes of 3 M sodium acetate pH 5.5 and 2.5 volumes 100% ethanol, and incubating at − 20 °C for 1 hr. followed by centrifugation at 12500 RPM for 20 minutes at 4 °C. The DNA pellets were washed twice with 70% ethanol, dried for 5 min at room temperature and resuspended in 40 μL of TE_0.1_ buffer (10 mM Tris-HCl, 0.1 mM EDTA pH 8). DNA quality was assessed by running 200 ng on a 0.8% agarose gel stained with ethidium bromide (0.5 μg/mL) in 1X TAE (Tris-acetate-EDTA) buffer. A total of 100 ng of DNA was used as input for PCR with the AM specific primers FLR3/FLR4 [74] carrying Illumina-compatible tails to screen root samples for the presence of AM. PCR reactions consisted of 1X Q5 buffer (New England Biolabs), 0.4 mM deoxynucleotide triphosphates (dNTPs), 0.5 μM of the AM-specific tailed forward primer FLR3 (5′-TCGTCGGCAGCGTCAGATGTGTATAAGAGACAGTTGAAAGGGAAACGATTGAAGT-3′), 0.5 μM of the AM-specific tailed reverse primer FLR4 (5′-GTCTCGTGGGCTCGGAGATGTGTATAAGAGACAGTACGTCAACATCCTTAACGAA-3′) [74], 0.5 U Q5 polymerase (New England Biolabs), 1 M betaine and 5% dimethyl sulfoxide (DMSO). PCR conditions consisted of an initial denaturation at 98 °C for 30s, 22 cycles of 98 °C for 15 s, 56 °C for 15 s and 72 °C for 20s, followed by a final extension at 72 °C for 10 min.

### MicroRNA identification

Adapters were trimmed from the reads obtained from the small RNA libraries and sequences 18–30 nucleotides were kept. Reads were aligned to the switchgrass reference genome version 5.0 (https://phytozome-next.jgi.doe.gov/info/Pvirgatum_v5_1) [40] with Bowtie version 1 [75] allowing no mismatches. Perfectly matched switchgrass reads were then aligned using the same parameters to the RFAM database version 12.3 [76] and reads with perfect alignment to rRNA, tRNA, snRNA and snoRNA were removed. The cleaned small RNA (sRNA) reads for each accession were combined across treatments and replicates into a single file. Duplicate reads were collapsed into a single representative read, and its abundance was recorded using the FastX toolkit “FASTQ/A Collapser” tool [77]. Using the collapsed read set, mature-miRNA-producing loci were predicted using miRDeep-P2 [78] with default parameters. Known miRNAs were identified by aligning the mature miRNA sequences to miRBase release 22 [79] using BlastN-short with a maximum of three mismatches. MiRNAs that were absent from miRBase but predicted in at least two genotypes were considered novel miRNAs.

### MicroRNA expression profiling

Cleaned reads from each miRNA library were realigned to the switchgrass version 5 genome sequence using Bowtie [75] with 0 mismatches. All valid alignments were retained and read counts corresponding to mature miRNA coordinates predicted by miRDeep-P2 [78] were obtained using the multicov tool from the bedtools package [80]. Raw read counts were analyzed with DESeq2 [81] to determine differential expression within each accession between AM and control conditions.

### MicroRNA target identification

Degradome sequence data were used to identify miRNA targets. Degradome reads were 3′ adapter trimmed and size selected for reads > 17 nt. MicroRNA directed cleavage signatures were identified using CleaveLand4 [82] with default parameters. In the CleaveLand4 output, degradome signatures are categorized on a scale from 0 (strongest degradome support) to 4 (weakest degradome support). Minimum free energy (MFE) ratios for miRNA-target duplexes were calculated using CleaveLand4 [82]. Putative miRNA targets that had an MFE ratio of 0.75 or higher and were identified as category 2 or lower in at least two degradome libraries were considered to have strong degradome support. Target-miRNA pairs that had an MFE ratio less than 0.75 required category 1 or 2 degradome signatures to be present in at least six degradome libraries to be considered strong candidate targets. Annotations for the degradome supported targets were obtained using the switchgrass mRNA sequence in a BLASTX search against the Swiss-Prot database (https://www.uniprot.org/).

All methods were performed in accordance with the relevant guidelines and regulations.

## Supplementary Information

**Additional file 1: Supplemental Table S1.** sRNA sequencing summary. **Supplemental Table S2.** miRNA prediction results separated by accession. **Supplemental Table S3.** miRNA precursor distribution across the switchgrass genome ordered by chromosome. **Supplemental Table S4.** miRNA precursor distribution across the switchgrass genome ordered by miRNA. **Supplemental Table S5.** AM-responsive miRNAs. **Supplemental Table S6.** Degradome validation summary. **Supplemental Table S7.** DEseq2 results for top 50 most differentially expressed miRNAs.

**Additional file 2: Supplemental Figure S1.** Length distribution of known and novel miRNAs.

**Additional file 3: Supplemental Figure S2.** Microscopic and PCR detection of AM in experimental groups.

## Data Availability

Raw small RNA and degradome sequencing data presented in this article can be found in NCBI under BioProject PRJNA740297 (https://dataview.ncbi.nlm.nih.gov/object/PRJNA740297?reviewer=rojc8lbs4itn4njndjjarfq1p). All other data generated during this study are included in the manuscript as Supplementary Information.
