# Supplementary figures and images for: Identification of microRNAs responsive to arbuscular mycorrhizal fungi in Panicum virgatum (switchgrass)
Source: BMC Genomics. 2022 Oct 5;23:688. doi: 10.1186/s12864-022-08797-x (PMC9535954; doi:10.1186/s12864-022-08797-x)

A

B

C


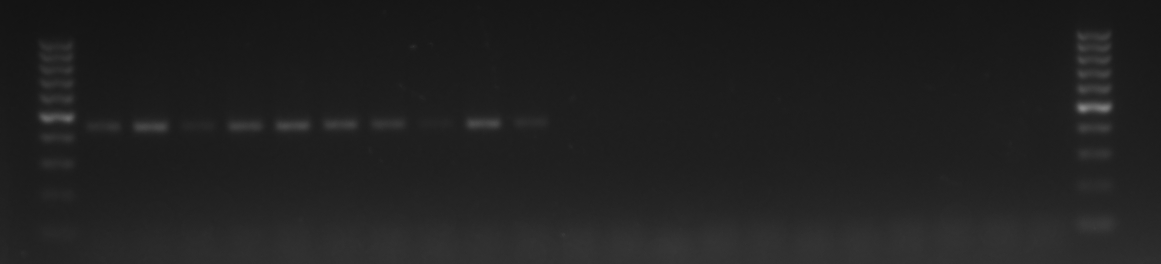

Supplement: Supplementary file 3 — Additional file 3: Supplemental Figure S2. Microscopic and PCR detection of AM in experimental groups. [file 12864_2022_8797_MOESM3_ESM.docx]
