# Supplementary material for: Identification of microRNAs responsive to arbuscular mycorrhizal fungi in Panicum virgatum (switchgrass)
Source: BMC Genomics. 2022 Oct 5;23:688. doi: 10.1186/s12864-022-08797-x (PMC9535954; doi:10.1186/s12864-022-08797-x)
Supplement: Supplementary file 3 [file 12864_2022_8797_MOESM2_ESM.docx]

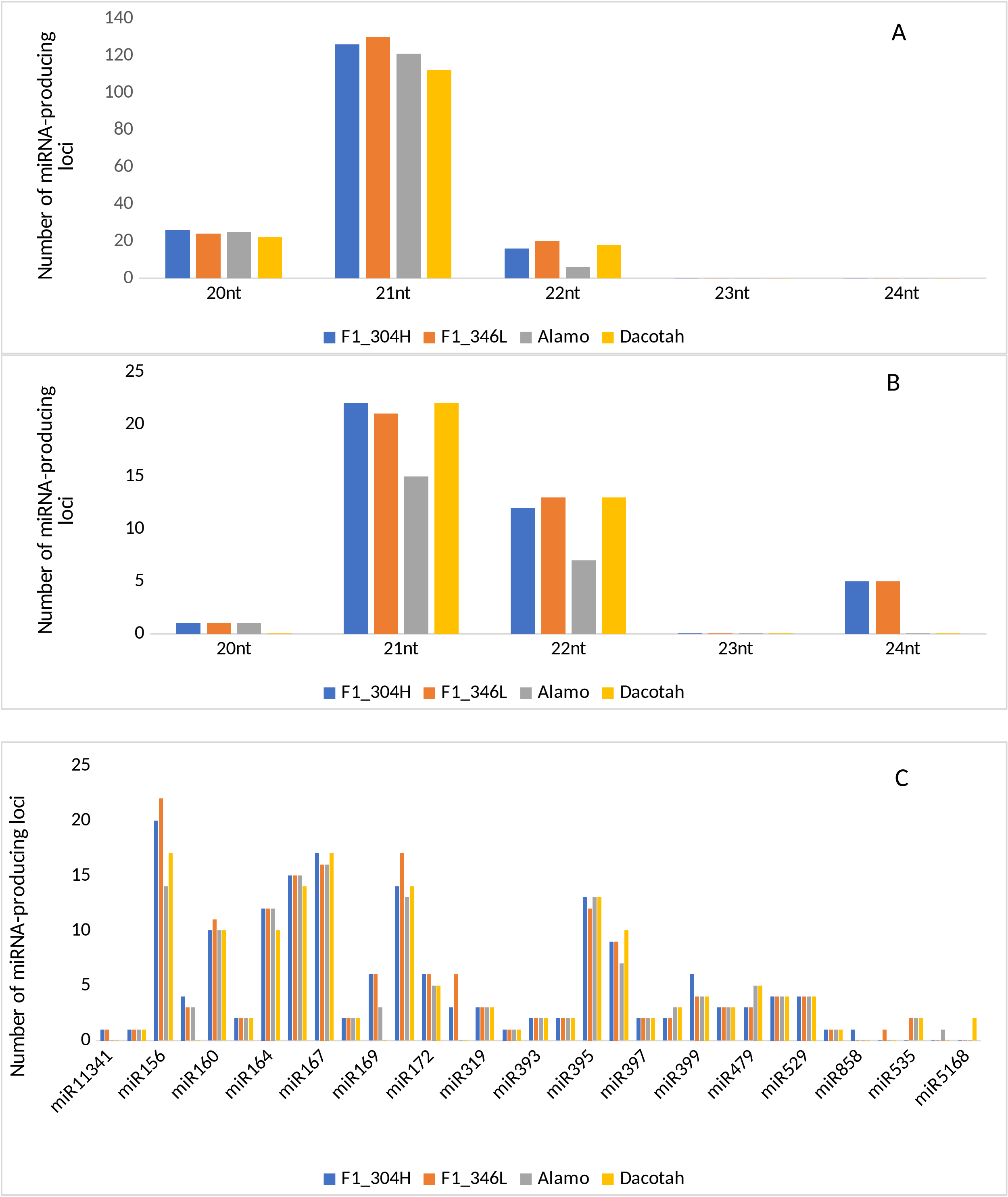

A
Number of miRNA-producing loci
140
120
100
80
60
40
20
0
20nt
21nt
22nt
23nt
24nt
F1_304H
F1_346L
Alamo
Dacotah
B
Number of miRNA-producing loci
25
20
15
10
5
0
20nt
21nt
22nt
23nt
24nt
F1_304H
F1_346L
Alamo
Dacotah
C
Number of miRNA-producing loci
25
20
15
10
5
0
miR11341
miR156
miR160
miR164
miR167
miR169
miR172
miR319
miR393
miR395
miR397
miR399
miR479
miR529
miR858
miR535
miR5168
F1_304H
F1_346L
Alamo
Dacotah
